# Supplementary material for: Individual differences in belief updating and phasic arousal are related to psychosis proneness
Source: Commun Psychol. 2024 Sep 23;2:88. doi: 10.1038/s44271-024-00140-2 (PMC11420346; doi:10.1038/s44271-024-00140-2)
Supplement: Supplementary file 2 — Supplementary Material [file 44271_2024_140_MOESM2_ESM.pdf]

Supplementary Information for "Individual Differences in Belief Updating and Phasic Arousal Are Related to Psychosis Proneness", *Communications Psychology*

Peter R Murphy[1,2,*], Katarina Krkovic[3], Gina Monov[1], Natalia Kudlek[1], Tania Lincoln[3†], Tobias H Donner[1,4†*]

[1]Section Computational Cognitive Neuroscience, Department of Neurophysiology and Pathophysiology, University Medical Center Hamburg-Eppendorf, Martinistrasse 52, Hamburg 20251, Germany

[2]Department of Psychology, Maynooth University, Co. Kildare, Ireland

[3]Department of Clinical Psychology and Psychotherapy, Institute of Psychology, University of Hamburg, Hamburg, Germany

[4]Bernstein Center for Computational Neuroscience, Charité Universitätsmedizin, Haus 6, Philippstraße 13, 10115, Berlin, Germany

*To whom correspondence should be addressed: t.donner@uke.de; peter.murphy@mu.ie

†These authors jointly supervised this work

Document contains:

Supplementary Table 1
Supplementary Figures 1-9

Supplementary Table 1. Summary of and motivation behind parameters of main behavioral task.

**Task parameter**	**Description**	**Chosen value**	**Motivation**
H	Probably of a change in generative state ('change-point') between successive samples	0.1	Produces regular change-points (65.1% of maximum length trials with 1 or more) while also encouraging periods of strong, stable beliefs. Within the range of *H* values identified in Murphy et al.[1] to produce strong change-point probability (*CPP*) and uncertainty (-|ψ|) modulations of evidence weighting in normative model.
SNR	Signal-to-noise ratio (difference in means divided by shared s.d.) of generative distributions	1.26	Sufficiently noisy to demand temporal integration across multiple samples but not noisy enough to preclude formation of strong beliefs. Within the range of *SNR* values identified in Murphy et al.[1] to produce strong *CPP* and -|ψ| modulations of evidence weighting in normative model.
Max. sequence length	Maximum number of evidence samples per trial	10	Sufficiently long to permit formation of strong, stable beliefs as well as occurrence of regular change-points given the chosen *H* and *SNR*. Short enough to permit collection of required number of trials per participant.
Number of trials	Number of trials per participant	1290-1376	Achievable given time constraints and shown via parameter recovery analyses to produce reliable estimates of model parameters.
P(max. sequence length)	Proportion of trials on which sequences of maximum length were presented	0.65	Yielded high number of maximum length trials per participant (839-894) for reliable estimation of individual psychophysical kernels.

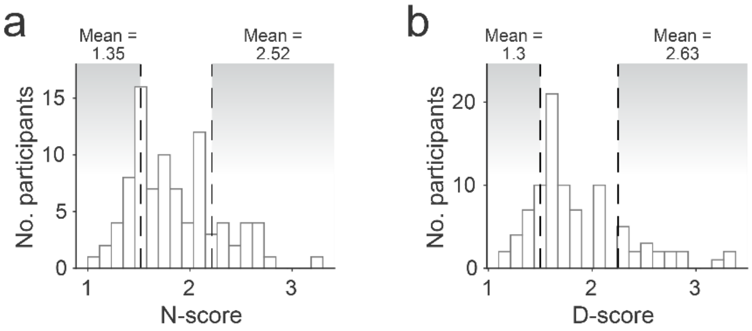

Supplementary Figure 1. Histograms of N- and D-scores from CAPE in our sample.

Like the P-scores shown in main Figure 1e, the N- and D-scores in the community sample also covered a range from healthy to values observed in ultra-high risk samples and in diagnosed patients[2-4].

*(**a**) Distribution of N-scores extracted from Community Assessment of Psychic Experiences (CAPE) questionnaire data (n=90 participants). Vertical dashed lines indicate cutoffs for lowest and highest N-score quintiles; means are mean N-scores within each sub-group (n=18 participants in each).*

*(**b**) Distribution of D-scores. Format same as in **a**.*

a

	Parameter			
	$\hat{H}$	Noise	B	IU
**Model 1**	X	X		
Model 2	X	X	X	
Model 3	X	X		X
Model 4	X	X	X	X

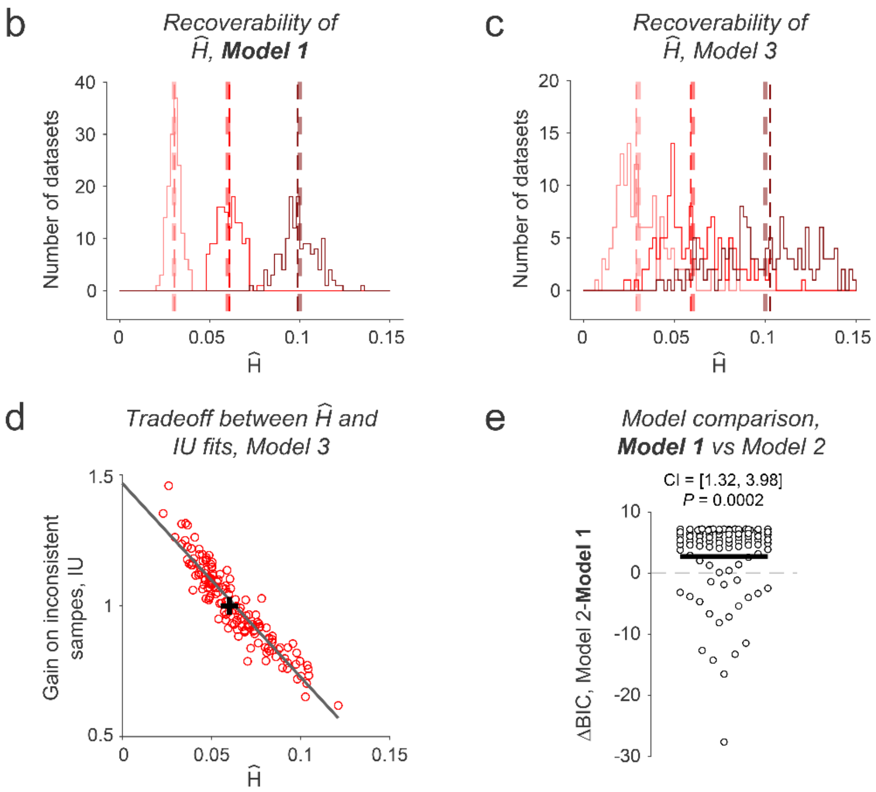

Supplementary Figure 2. Model validation and comparison favor normative belief updating model with two free parameters, $\hat{H}$ and decision noise.

*(**a**) Table specifying free parameters included in each considered model variant. See Methods for descriptions of individual parameters. The model variant reported in the main text, Model 1, is highlighted in bold throughout the figure.*

*(**b**) Recoverability of the $\hat{H}$ parameter from fits of Model 1. 150 datasets were generated with noise = 2 (close to the group mean value in model fits to our human participants) and $\hat{H}$ set to each of three representative levels (0.03, 0.06 and 0.1; thick vertical dashed lines). Histograms show distributions of recovered $\hat{H}$ parameters (thin vertical dashed lines indicate median $\hat{H}$ per generative parameter set)*

*(**c**) Same as **b**, but now from fits of Model 3 (which includes a gain on inconsistent evidence parameter, IU, as an additional free parameter). Note the significant increase in the variability of the recovered $\hat{H}$ parameters relative to fits of Model 1 in **b**.*

*(**d**) Correlation between $\hat{H}$ and IU estimated from fits of Model 3. Cross indicates true generative parameters. Strong negative correlation reflects a tradeoff in fitted $\hat{H}$ and IU parameters, highlighting parameter recovery issue caused by introduction of IU as an additional free parameter. Thus Models 3 and 4 were not considered further.*

*(**e**) Difference in Bayes Information Criterion (BIC) values for fits of Models 1 and 2 to the participants' data (n=90). Positive values indicate support for Model 1, negative values support for Model 2. Although Model 2 provided a better quantitative fit for a modest subset of participants (19 participants with negative values in plot), Model 1 provided the better fit at the group level (P-value, two-tailed permutation test comparing Model 1 and Model 2 BICs). CI, 95% confidence intervals (bootstrapped) around mean BIC difference.*

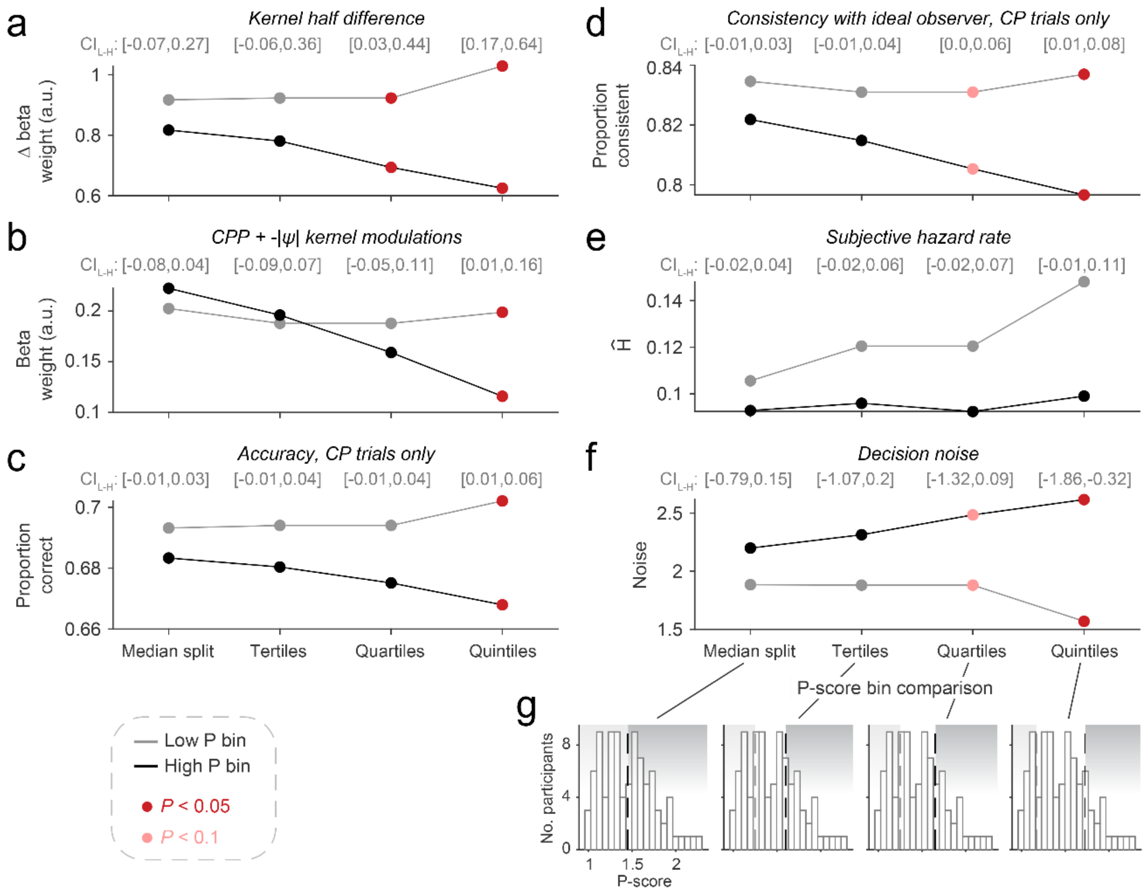

Supplementary Figure 3. Effect of participant binning on relationships of evidence weighting, behavioral measures and model parameters with individual differences in P-scores.

Each plot charts across-participant means of a behavioral or model-based measure, for low (grey) and high (black) P-score participant subgroups defined by each of four different binning procedures (from left to right: median split producing n=45 participants per subgroup, first vs. third tertiles producing n=30 per subgroup, first vs. fourth quartiles producing n=23 per subgroup, first vs. fifth quintiles producing n=18 per subgroup). Colored markers highlight statistically significant (dark red, P<0.05) or marginally significant (light red, P<0.1) effects of P-score subgroup for the corresponding binning procedure (two-sample permutation test). $CI_{L\text{-}H}$, 95% confidence intervals (bootstrapped) around difference of means between subgroups with lowest and highest P-scores.

*(**a**) Kernel half-difference (subtraction of mean weighting of first 5 samples from mean weighting of last 5 samples) capturing degree of recency in evidence weighting*

*(**b**) Summed strength of modulations of evidence weighting by CPP (mean modulation weights over sample positions 4-10, significant cluster in Figure 2d) and -|ψ| (mean modulation weights over sample positions 3-8, significant cluster in Figure 2e).*

*(**c**) Choice accuracy on trials with at least one change-point in task generative state.*

*(**d**) Consistency of participant choices with those of the ideal observer on trials with at least one change-point in task generative state.*

*(**e**) Subjective hazard rate parameters from fits of the normative model to participants' choices.*

*(**f**) Decision noise parameters from fits of the normative model to participants' choices.*

*(**g**) Histogram of P-scores from entire sample with cutoffs for low and high P subgroups highlighted for each of the four binning procedures.*

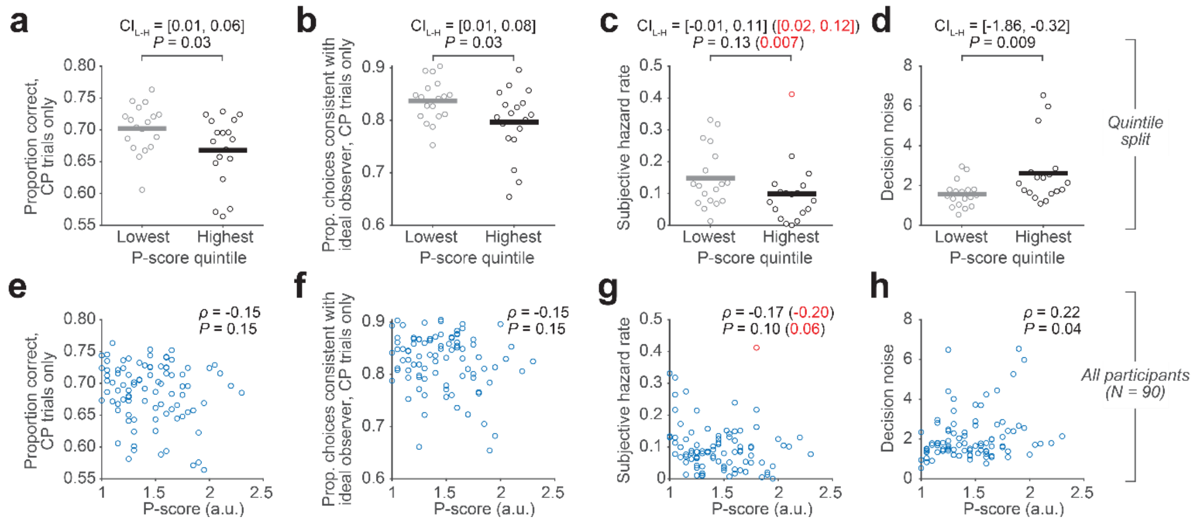

Supplementary Figure 4. Relationship of overall performance and fitted model parameters with individual differences in P-scores.

*(**a**) Choice accuracy on trials with at least one change-point in task generative state, plotted for participants in lowest and highest P-score quintiles (n=18 participants per quintile subgroup). Horizontal lines, mean of data from each subgroup; circles, individual participants. P-value, two-sample permutation test (two-tailed). CI_{L-H}, 95% confidence intervals (bootstrapped) around difference of means between lowest and highest P-score quintile subgroups.*

*(**b**) Consistency of participant choices with those of the ideal observer on trials with at least one change-point in task generative state. Same layout as in **a**.*

*(**c**) Subjective hazard rate parameters from fits of the normative model to participants' choices. Same layout as in **a**,**b**.*

*(**d**) Decision noise parameters from fits of the normative model to participants' choices. Same layout as in **a**-**c**.*

*(**e-h**) Same performance measures and fitted model parameters as in **a**-**d** but now plotted in scatterplots against P-scores and including all participants (circles; n=90). Correlation coefficients and P-values, Spearman correlation.*

*Single participant highlighted in red in **c**,**g** is a potential outlier in the distribution of subjective hazard rate fits. Statistics in parenthesis and red font are from analyses (including re-running of quintile binning, not shown) with this participant excluded.*

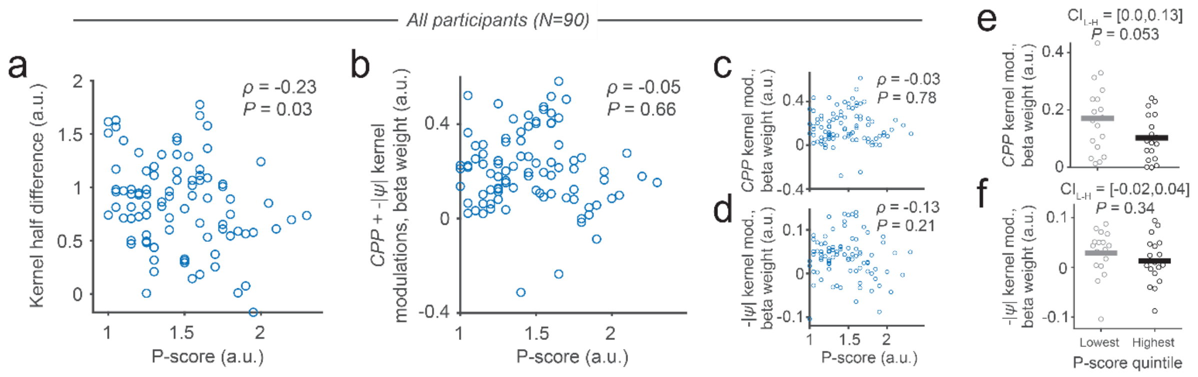

Supplementary Figure 5. Correlations between kernel metrics and P-scores across all participants, and decomposition of summed *CPP* and -|ψ| quintile bin effect.

*(**a**) Scatterplot for entire sample (n=90 participants) of P-scores against kernel half-difference for the overall evidence weighting profile.*

*(**b**) Scatterplot of P-scores against summed strength of modulations of evidence weighting by CPP (mean modulation weights over sample positions 4-10, significant cluster in Figure 2d) and -|ψ| (mean modulation weights over sample positions 3-8, significant cluster in Figure 2e).*

*(**c**,**d**) Scatterplots of P-scores against individual CPP (**c**) and -|ψ| (**d**) modulations.*

*(**e**,**f**) Individual CPP (**e**) and -|ψ| (**f**) modulations for lowest and highest P-score quintiles (n=18 participants per quintile subgroup).*

*Correlation coefficients and P-values in **a**-**d**, Spearman correlation (n=90 participants). P-values in **e**,**f**, two-sample permutation tests (two-tailed). CI_{L-H} in **e**,**f**, 95% confidence intervals (bootstrapped) around difference of means between lowest and highest P-score subgroups (n=18 participants per subgroup).*

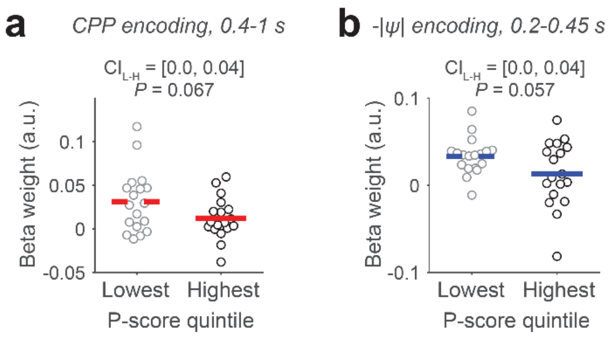

Supplementary Figure 6. Pupil encoding of change-point probability (*CPP*) and uncertainty (-|ψ|) in high and low P-score subgroups.

*(**a**) CPP encoding. (**b**) Uncertainty encoding. The time windows for each variable were chosen based on the significant encoding in the time-resolved analysis of the whole group (Figure 4d). For both variables, differences between subgroups were marginally significant (P-values, permutation tests). CI_{L-H}, 95% confidence intervals (bootstrapped) around difference of means between subgroups with lowest and highest P-scores (n=18 participants per subgroup).*

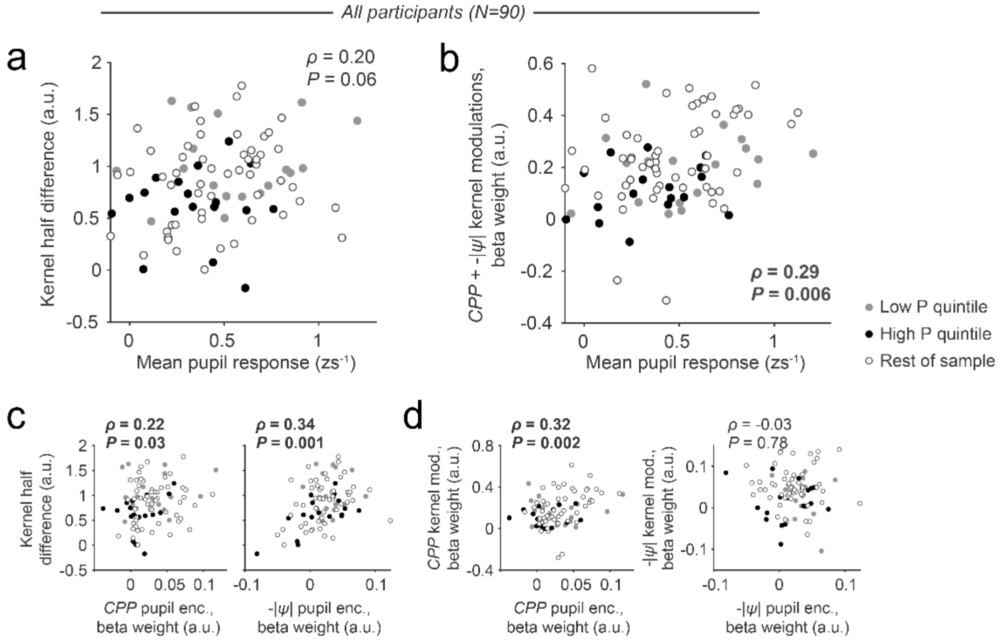

Supplementary Figure 7. Correlations between kernel and pupil metrics across all participants.

*(**a**) Scatterplot of the mean pupil derivative response (0.2-1 s following trial onset) against the kernel half-difference measure derived from the overall evidence weighting profile.*

*(**b**) Mean response of the pupil derivative plotted against summed magnitude of CPP (sample positions 4-10) and -|ψ| (positions 3-8) modulations of evidence weighting.*

*(**c**) Decomposition of correlation reported in Figure 5a, separately plotting kernel half-difference against pupil encoding of both CPP and -|ψ|.*

*(**d**) Decomposition of correlation reported in Figure 5b, separately plotting CPP modulation of evidence weighting against pupil encoding of CPP, and -|ψ| modulation of evidence weighting against pupil encoding of -|ψ|.*

Circles, individual participants (n=90); circle colours, low P subgroup (light grey, filled), high P subgroup (black, filled) and remainder of sample (grey, unfilled). Correlation coefficients and P-values, Spearman correlation. Statistically significant (P<0.05) correlations highlighted in bold text.

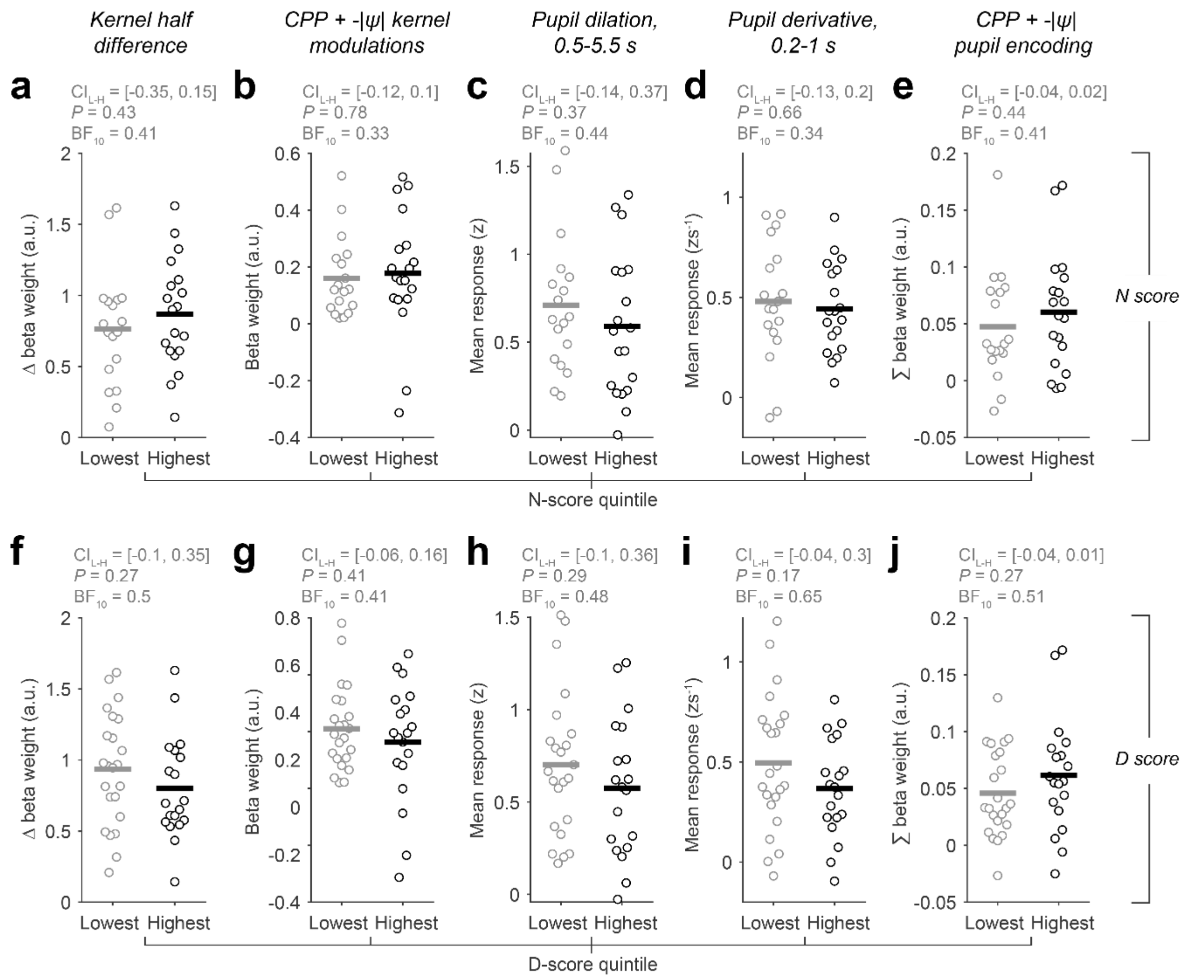

Supplementary Figure 8. Relationships of evidence weighting and pupil measures to individual differences in N and D scores measured via the CAPE.

*(**a**) Kernel half-difference summary measure (subtraction of mean weighting of first 5 samples from mean weighting of last 5 samples) capturing degree of recency in evidence weighting, plotted for participant subgroups defined by lowest and highest N-score quintiles.*

*(**b**) Summed strength of modulations of evidence weighting by CPP (mean modulation weights over sample positions 4-10, significant cluster in Figure 2d) and -|ψ| (mean modulation weights over sample positions 3-8, significant cluster in Figure 2e), plotted for lowest and highest N-score quintiles.*

*(**c**) Overall trial-related pupil response for lowest and highest N-score quintiles.*

*(**d**) Early response of the pupil first derivative for lowest and highest N-score quintiles.*

*(**e**) Encoding of change-point probability (CPP) and uncertainty (-|ψ|) in pupil responses (pooled) for lowest and highest N-score quintiles.*

*(**f-j**) Same format and dependent variables as **a**-**e**, but now with participant subgroups defined by lowest and highest quintiles of the D-score distribution.*

Horizontal lines in all panels, mean of data from each subgroup; circles, individual participants (n=18 per subgroup). P-values, two-sample permutation tests (two-tailed). CI_{L-H}, 95% confidence intervals (bootstrapped) around difference of means between quintile subgroups with lowest and highest questionnaire scores. BF_{10}, Bayes factors capturing relative strength of evidence for an effect of subgroup versus no effect.

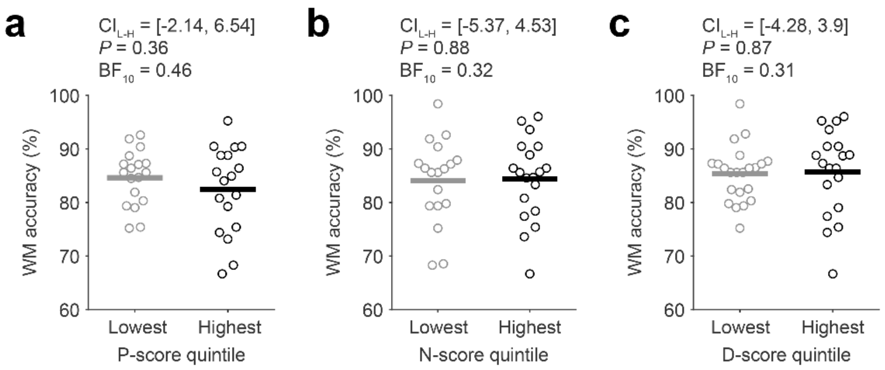

Supplementary Figure 9. Relationships of CAPE scores to performance on delayed match-to-sample working memory task.

*(**a**) Accuracy of '*same*'/'*different*' reports on delayed match-to-sample working memory task, plotted for participant subgroups defined by lowest and highest P-score quintiles. See Methods for details on delayed match-to-sample task.*

*(**b**) Same as **a**, but now with participant subgroups defined by lowest and highest quintiles of the N-score distribution.*

*(**c**) Same as **a,b**, but now with participant subgroups defined by lowest and highest quintiles of the D-score distribution.*

Horizontal lines in all panels, mean of data from each subgroup; circles, individual participants (n=18 per subgroup). P-values, two-sample permutation tests (two-tailed). CI_{L-H}, 95% confidence intervals (bootstrapped) around difference of means between subgroups with lowest and highest P-scores. BF_{10}, Bayes factors capturing relative strength of evidence for an effect of subgroup versus no effect.

Supplementary References

1 Murphy, P. R., Wilming, N., Hernandez-Bocanegra, D. C., Prat-Ortega, G. & Donner, T. H. Adaptive circuit dynamics across human cortex during evidence accumulation in changing environments. *Nat. Neurosci.* **24**, 987-997, doi:10.1038/s41593-021-00839-z (2021).

2 Jaya, E. S. *et al.* The Community Assessment of Psychic Experiences: Optimal cut-off scores for detecting individuals with a psychotic disorder. *Int. J. Methods Psychiatr. Res.* **30**, e1893, doi:10.1002/mpr.1893 (2021).

3 Mossaheb, N. *et al.* The Community Assessment of Psychic Experience (CAPE) questionnaire as a screening-instrument in the detection of individuals at ultra-high risk for psychosis. *Schizophr. Res.* **141**, 210-214, doi:10.1016/j.schres.2012.08.008 (2012).

4 Schlier, B., Jaya, E. S., Moritz, S. & Lincoln, T. M. The Community Assessment of Psychic Experiences measures nine clusters of psychosis-like experiences: A validation of the German version of the CAPE. *Schizophr. Res.* **169**, 274-279, doi:10.1016/j.schres.2015.10.034 (2015).
