# Supplementary material for: Individual differences in belief updating and phasic arousal are related to psychosis proneness
Source: Commun Psychol. 2024 Sep 23;2:88. doi: 10.1038/s44271-024-00140-2 (PMC11420346; doi:10.1038/s44271-024-00140-2)
Supplement: Supplementary file 3 — Reporting summary [file 44271_2024_140_MOESM3_ESM.pdf]

nature portfolio

Corresponding author(s): Peter Murphy, Tobias Donner

Last updated by author(s): Aug 30, 2024

Reporting Summary

Nature Portfolio wishes to improve the reproducibility of the work that we publish. This form provides structure for consistency and transparency in reporting. For further information on Nature Portfolio policies, see our Editorial Policies and the Editorial Policy Checklist.

Statistics

For all statistical analyses, confirm that the following items are present in the figure legend, table legend, main text, or Methods section.

n/a	Confirmed	
☐	☒	The exact sample size (*n*) for each experimental group/condition, given as a discrete number and unit of measurement
☐	☒	A statement on whether measurements were taken from distinct samples or whether the same sample was measured repeatedly
☐	☒	The statistical test(s) used AND whether they are one- or two-sided<br>*Only common tests should be described solely by name; describe more complex techniques in the Methods section.*
☐	☒	A description of all covariates tested
☐	☒	A description of any assumptions or corrections, such as tests of normality and adjustment for multiple comparisons
☐	☒	A full description of the statistical parameters including central tendency (e.g. means) or other basic estimates (e.g. regression coefficient) AND variation (e.g. standard deviation) or associated estimates of uncertainty (e.g. confidence intervals)
☐	☒	For null hypothesis testing, the test statistic (e.g. *F*, *t*, *r*) with confidence intervals, effect sizes, degrees of freedom and *P* value noted<br>*Give P values as exact values whenever suitable.*
☐	☒	For Bayesian analysis, information on the choice of priors and Markov chain Monte Carlo settings
☒	☐	For hierarchical and complex designs, identification of the appropriate level for tests and full reporting of outcomes
☐	☒	Estimates of effect sizes (e.g. Cohen's *d*, Pearson's *r*), indicating how they were calculated

Our web collection on statistics for biologists contains articles on many of the points above.

Software and code

Policy information about availability of computer code

Data collection	Eye-tracking and pupillometry data were collected using the acquisition software developed by the system manufacturer (SMI iViewX). Behavioral data were collected using Matlab version 2018b, using stimulus presentation functions from Psychtoolbox 3.
Data analysis	Matlab version 2021a was used for all analyses, including custom code (behavioral analysis, modelling, pupil analysis) and the Particle Swarm Optimization toolbox version 1.0.0.0 (model fitting). All custom code is publicly available at https://github.com/murphyp7/2024_Murphy_Belief-updating-psychosis-proneness.

For manuscripts utilizing custom algorithms or software that are central to the research but not yet described in published literature, software must be made available to editors and reviewers. We strongly encourage code deposition in a community repository (e.g. GitHub). See the Nature Portfolio guidelines for submitting code & software for further information.

Data

Policy information about availability of data

All manuscripts must include a data availability statement. This statement should provide the following information, where applicable:

- Accession codes, unique identifiers, or web links for publicly available datasets
- A description of any restrictions on data availability
- For clinical datasets or third party data, please ensure that the statement adheres to our policy

Raw demographic, questionnaire, behavioral and eye-tracking data, and the data presented in all 14 figures across main text and supplement, are publicly available

at the following open access repository: http://doi.org/10.25592/uhhfdm.14759. We include a data availability statement indicating this in our manuscript, and provide the link to the repository.

Human research participants

Policy information about studies involving human research participants and Sex and Gender in Research.

Reporting on sex and gender	Only data on biological sex were gathered, via self-report. Of the 90 participants included in our analyzed sample, 48 self-reported to be female. This information is reported in the 'Recruitment and Sample' subsection of the Methods section in the manuscript and will be shared in anonymized but disaggregated form as part of the source data prior to publication. Sex and gender were not explicitly considered as part of the study design. Sex-based analyses were not performed as they were not deemed to be of central importance to the research questions that we sought to address with this manuscript, and the same analyses that we report here conducted separately for each sex risk being underpowered.
Population characteristics	We report age and self-reported past diagnosis information pertaining to our current sample. The mean ± s.d. age was 31.6 ± 9.5 years (range 18-55). Four participants self-reported a previous diagnosis of depression, one of emotionally unstable personality disorder (impulsive type), one of post-traumatic stress disorder (PTSD), one of obsessive-compulsive disorder (OCD), one of comorbid depression and attention-deficit hyperactivity disorder (ADHD), and one of psychosis due to substance abuse.
Recruitment	Participants were recruited through advertising in the locality (community centers, university message boards, etc). Given the skewness of P-scores (our measure of psychosis proneness) in the population, with the majority scoring at the low end and few scoring at the higher end of the psychosis continuum, we oversampled participants in the high range aiming for 50% with a P-score above the 50th percentile within a large community sample. This process is described in detail in the Methods section.
Ethics oversight	The study was approved by the ethics committee of the Faculty of Psychology and Movement Science at Universität Hamburg.

Note that full information on the approval of the study protocol must also be provided in the manuscript.

Field-specific reporting

Please select the one below that is the best fit for your research. If you are not sure, read the appropriate sections before making your selection.

☒ Life sciences ☐ Behavioural & social sciences ☐ Ecological, evolutionary & environmental sciences

For a reference copy of the document with all sections, see nature.com/documents/nr-reporting-summary-flat.pdf

Life sciences study design

All studies must disclose on these points even when the disclosure is negative.

Sample size	No statistical methods were used to pre-determine sample size but our sample size of 90 is equal to or larger than those reported in previous publications for comparable designs (e.g. Browning et al., 2015, Nature Neuroscience). Of particular note, each of our 90 participants attended 2 sessions and we collected between 1290-1376 analyzable trials per participant, which is unusually large relative to existing studies and facilitates meaningful data analysis at the level of single subjects (e.g. Figure 2a of main manuscript). Prior to data collection, we also verified through simulations of the normative model for our task that this combination of sample size and trial counts were more than sufficient to produce critical behavioral effects (modulation of evidence weighting by change-point probability and uncertainty) even for high levels of internal noise.
Data exclusions	Four participants were excluded from all analyses due to failing to complete both testing sessions, and 2 more participants were excluded due to incomplete CAPE questionnaire responses. An additional 2 participants were excluded from analyses of data from the delayed match-to-sample task having failed to complete any blocks of this task due to time constraints. These issues were not anticipated and so corresponding exclusion criteria were not specified in advance.<br>At the single subject level, we used pre-established criteria based on previous studies from our lab to exclude single trials in which eye-tracking data were contaminated by well-known artifacts (e.g. by blinks during the stimulus sequence).
Replication	Key effects reported in Figures 2c-e (evidence weighting profiles) and 4a,d constitute replications of effects that we reported previously (Murphy et al., 2021, Nature Neuroscience) under different task generative statistics. All other reported results are novel effects that we do not attempt to replicate in the current manuscript.
Randomization	Subjects were all allocated into a single group for many analyses that we report; and for other analyses, subsets of subjects were allocated posthoc to each of two groups on the basis of their P-scores. As such, randomization of subjects to experimental groups was not necessary.
Blinding	As described above, the only analyses that involved comparison of groups of subjects was when subsets of subjects were allocated posthoc to each of two non-experimental groups on the basis of their P-scores. As such, blinding was not necessary.

Reporting for specific materials, systems and methods

We require information from authors about some types of materials, experimental systems and methods used in many studies. Here, indicate whether each material, system or method listed is relevant to your study. If you are not sure if a list item applies to your research, read the appropriate section before selecting a response.

Materials & experimental systems

n/a	Involved in the study
☒	☐ Antibodies
☒	☐ Eukaryotic cell lines
☒	☐ Palaeontology and archaeology
☒	☐ Animals and other organisms
☒	☐ Clinical data
☒	☐ Dual use research of concern

Methods

n/a	Involved in the study
☒	☐ ChIP-seq
☒	☐ Flow cytometry
☒	☐ MRI-based neuroimaging
